# Excitation-emission fluorescence matrix acquired from glutathione capped CdSeS/ZnS quantum dots in combination with chemometric tools for pattern-based sensing of neurotransmitters

**DOI:** 10.1007/s00604-021-04984-x

**Published:** 2021-09-15

**Authors:** Klaudia Głowacz, Marcin Drozd, Patrycja Ciosek-Skibińska

**Affiliations:** 1 Chair of Medical Biotechnology, Faculty of Chemistry, Warsaw University of Technology, Noakowskiego 3, 00-664 Warsaw, Poland; 2 Centre for Advanced Materials and Technologies CEZAMAT, Poleczki 19, 02-822 Warsaw, Poland

**Keywords:** Pattern-based sensing, Excitation-emission matrix, 2D fluorescence, Quantum dots, Neurotransmitters

## Abstract

The presented work concerns pattern-based sensing with quantum dots for the identification and quantification of neurotransmitters by means of excitation-emission fluorescence spectroscopy (2D fluorescence). In the framework of this study, glutathione capped CdSeS/ZnS nanocrystals were used as non-specific nanoreceptors capable of differentiated interaction with neurotransmitters. The pattern-based sensing with QDs was realized by using excitation-emission fluorescence spectroscopy to provide analyte-specific multidimensional optical information. These characteristic fluorescent response patterns were processed by unfolded partial least squares–discriminant analysis, showing that satisfactory identification of all investigated neurotransmitters: dopamine, norepinephrine, epinephrine, serotonin, GABA, and acetylcholine, can be achieved through the proposed sensing strategy. The impact of the considered fluorescence signal (datum, i.e. zeroth-order data acquired per sample; spectrum, i.e. first-order data acquired per sample; excitation-emission matrix, i.e. second-order data acquired per sample) on the sensing capability of glutathione capped QDs was also verified. The best performance parameters such as accuracy, precision, sensitivity, and specificity were obtained using excitation-emission matrices (88.9–93.3%, 0.93–0.95, 0.89–0.93, and 0.99–1.00, respectively). Thus, it was revealed that excitation-emission fluorescence spectroscopy may improve the recognition of neurotransmitters while using only one type of nanoreceptor. Furthermore, is was demonstrated that the proposed excitation-emission fluorescence spectroscopy assisted QD assay coupled with unfolded partial least squares regression can be successfully utilized for quantitative determination of catecholamine neurotransmitters at the micromolar concentration range with *R*^2^ in the range 0.916–0.987. Consequently, the proposed sensing strategy has the potential to significantly simplify the sensing element and to expand the pool of bioanalytes so far detectable with the use of QDs.

**Graphical abstract:**

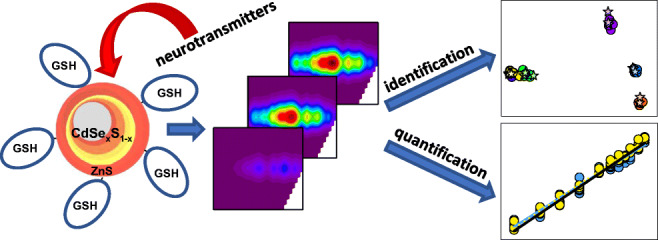

**Supplementary Information:**

The online version contains supplementary material available at 10.1007/s00604-021-04984-x.

## Introduction

Semiconductor quantum dots (QDs) are widely used in many fields of bioanalysis, such as bioimaging [1, 2] and theranostics [3], due to their unique optical properties. They are manifested by high photostability, narrow, sharp, symmetric emission spectra, wide absorption spectra, relatively high quantum yields, and generally long photoluminescence decay times, which arise from quantum confinement combined with large biochemically accessible surface [4]. Due to the small size of QDs and thus high surface-to-volume ratio as well as the photoluminescence (PL) mechanism itself [5], any subtle interactions between surface atoms and the surrounding molecules can significantly influence QDs’ photoluminescence characteristics such as quenching/enhancement of emission intensity, spectral shifts, and change in the PL decay time, which make them one of the most advantageous nanoparticles for chemical sensing. Therefore, it is not surprising that over the years, quantum dots have been used as nanosensors for the detection of various biologically relevant analytes [4, 6]. However, typically detection relies on a specific lock-and-key recognition mechanism that may require chemical modification or surface functionalisation, followed by conjugation with a biomolecules of strictly defined biological significance [1]. This approach not only can be quite demanding and time-consuming, but also does not take advantage of variety of non-specific interactions (e.g. electrostatic interactions, van der Waals forces, and hydrogen-bonding interactions) affecting QDs’ fluorescent response that can be utilized in pattern-based sensing [7].

The concept of pattern-based sensing (also called ‘chemical noses’ or ‘differential sensing’) is simply to design receptors that differentially interact with various analyte components [8]. Therefore, a strong affinity of analyte for one particular receptor is not required, because recognition is achieved by the composite response of entire array of receptors, where each receptor within the array may bind to multiple analytes, but recognize each of them to varied extents. As a result, a composite fingerprint is obtained that provides characteristic patterns for the individual analyte or complex mixtures comprised of multiple analytes [9, 10]. Quantum dots seem to be ideal candidates for the development of pattern-based sensing platforms due to the ease of modulation of their physicochemical properties. The proper selection of the core material as well as functionalization of the QD surface may allow for obtaining nanoparticles with varied affinities to various chemical targets [7, 11, 12]. Although ‘chemical nose’ approach originally involves the use of several receptors to generate a multidimensional response, it can also be realized by using one type of nanoparticle with multifunctional surface modification [13] or by monitoring the effect of non-specific interactions on the optical properties of the nanoreceptor in different detection modes [14] for the production of multidimensional optical information.

In our previous work [15], we showed that the pattern-based sensing with one type of QDs employing non-specific interactions can be achieved by using excitation-emission matrix (EEM) fluorescence spectroscopy, which is most commonly used in the ‘fingerprint’ analysis of foodstuff [16], environmental samples [17], and monitoring of biotechnological processes [18]. Although EEM spectroscopy was originally proposed as a technique allowing for complex characterization of samples containing many fluorophores [18], it has been shown that it can also be a useful tool for the characterization of nanomaterials [19, 20], studying the interactions of QDs with biomolecules [21], as well as detection technique for QDs-based nanosensors [22, 23].

In this work, we further explore the potential of excitation-emission fluorescence spectroscopy (2D fluorescence) assisted pattern-based sensing with quantum dots. For this purpose, glutathione capped CdSeS/ZnS nanocrystals (QDs-GSH) prepared by ligand exchange method were used for recognition and quantification of neurotransmitters (dopamine, norepinephrine, epinephrine, serotonin, γ-aminobutyric acid–GABA, acetylcholine) by means of chemometric tools. Since the glutathione molecule contains functional groups of different chemical nature, which might be capable of differentiated interactions with various bioanalytes, its cross-affinity towards selected neurotransmitters with different structures was utilized. Moreover, in order to confirm the suitability of EEM spectroscopy for identification of bioanalytes on the basics of non-specific interactions, we also investigated how the type of fluorescence response (datum, 0D, i.e. zeroth-order data acquired per sample; spectrum, 1D, i.e. first-order data acquired per sample; excitation-emission matrix, 2D, i.e. second-order data acquired per sample) affects the sensing capability of QDs-GSH.

## Experimental

### Reagents and materials

Oleic acid-capped quantum dots (QDs-OA) with alloyed (CdSe_x_S_1-x_) core and ZnS shell (CdSeS/ZnS), nanocrystal diameter = 6.0 nm, λ_em_ = 630 nm, 1 mg/mL in toluene were purchased from Cytodiagnostics Inc. (Burlington, Canada). Glutathione in reduced form (GSH) was from Acros Organics (Geel, Belgium). Tetramethylammonium hydroxide pentahydrate (TMAH) was from Alfa Aesar (Kandel, Germany). Chloroform, anhydrous ethanol, and phosphoric acid (85%) were obtained from Chempur (Piekary Śląskie, Poland). Dialysis tubing cellulose membrane (MWCO = 12 kDa), dopamine hydrochloride, L-epinephrine, L-norepinephrine, serotonin hydrochloride, γ-aminobutyric acid (GABA), acetylcholine chloride, sodium phosphate monohydrate, disodium phosphate dodecahydrate, and sodium hydroxide were supplied by Sigma-Merck (Poznań, Poland). Milli-Q water was used for preparation of all aqueous solutions. All chemicals were used as received.

### Preparation of GSH capped quantum dots

Glutathione-capped CdSeS/ZnS quantum dots (QDs-GSH) were prepared by biphasic ligand exchange of commercially available, alloyed-type QDs according to procedures previously established for other thiolate ligands and described by Tamang et al. and Liu et al. [24, 25]. In brief, oleic acid-capped nanocrystals (2 mL, 1 mg/mL QDs solution in toluene) were pre-purified by precipitation with anhydrous ethanol (8 mL) followed by centrifugation in Falcon tube (RCF = 16,854 × g, 15 min). After separation, the pellet was reconstituted in chloroform (8 mL). In parallel, glutathione aqueous solution (8 mL, 100 mM) was prepared. The pH value of GSH aqueous solution was adjusted to pH 11.4 with 5 M TMAH, and the ligand solution was briefly purged with nitrogen. Freshly prepared, alkaline solution of reduced GSH (8 mL) was combined with QDs suspended in toluene (8 mL) resulting in formation of a biphasic, organic/aqueous system. Ligand exchange process was carried out in tightly sealed and vigorously shaken glassy vial, under nitrogen atmosphere and in the absence of light for 3 h. Finalization of biphasic ligand exchange process (represented by the disappearance of fluorescence in the bottom, organic phase) was controlled visually, under UV light. Then, the transparent, organic phase was discarded. The entire aqueous fraction (~ 8 mL) was purified by triplicate dialysis against 2 L of 1 mM phosphate/NaOH buffer pH 7.4. The first dialysis lasted 2 h; the next two were carried out overnight. The effectiveness of QDs purification was confirmed by measuring the pH of QDs solution, which should be equal to the pH of the dialysate (~ pH = 7.4). Quantum dots modified in this way were stored in a tightly sealed vial at 4 °C, protected from light and can be used for current research for at least 4 weeks after preparation.

### Characterization techniques and fluorescence measurements

UV-Vis absorption spectra of QDs were measured using Lambda 25 spectrophotometer (Perkin Elmer, Inc., Waltham, MA, USA) in quartz cuvettes of volume = 3.5 mL and path length = 1 cm (Hellma GmbH & Co, Müllheim, Germany) at room temperature. The concentration of QDs-GSH after dialysis was determined based on the absorbance at the first excitation according to the commonly used protocol described by Yu et al. [26]. Due to the alloyed-type of QD cores, the determination was comparative and based on the relative absorbance of excitonic peaks—the solution of QDs in toluene (1 mg/mL) was used as a reference. Dynamic light scattering (DLS) measurements of hydrodynamic diameter and ζ-potential of QDs-GSH were carried out at 25 °C by means of Zetasizer Nano ZS (Malvern Panalytical Ltd., Malvern, UK) using disposable, polystyrene cuvettes, and dip cell for ζ-potential measurements. For the comparative characterization of the fluorescence spectra of QDs before and after ligand exchange, FluoroMax®-3 spectrofluorimeter (Horiba Jobin Yvon, Longjumeau Cedex, France) and fluorescence quartz cuvettes of volume = 3.5 mL and path length = 1 cm (Hellma GmbH & Co, Müllheim, Germany) were used. All remaining fluorescence measurements of QDs-GSH including zeroth-order fluorescence signal (0D, i.e. fluorescence intensity at specific excitation/emission wavelength) and first-order fluorescence signal (1D, i.e. fluorescence spectrum at specific excitation wavelength), as well as excitation-emission matrixes (2D, second-order fluorescence data, i.e. fluorescence matrix acquired per sample at several excitation/emission wavelengths) were recorded by Synergy 2 Multi-Mode Reader fluorescence spectrometer (BioTek Instruments, Inc., Winooski, VT, USA), using Polystyrene Greiner CELLSTAR® 96-well plates (Greiner Bio-One GmbH, Kremsmünster, Austria). The detailed description of measurement protocol used for excitation-emission matrix acquisition is available in Supplementary Information (Table S.1).

### The detection of neurotransmitters

For the qualitative analysis of neurotransmitters (NTs), a 96-well plate was prepared as follows: the solution of QDs-GSH was pipetted to each well and diluted with 1 mM phosphate buffer at pH 7.4 to the volume of 190 μL. Then, 10 μL of neurotransmitter solution was added, so that the final concentration of QDs-GSH and bioanalyte was 0.01 mg/mL and 50 μM, respectively. This procedure was repeated for each of six investigated bioanalytes (dopamine, norepinephrine, epinephrine, serotonin, GABA, acetylcholine). The control samples of quantum dots were also considered, where instead of 10 μL of NT solution, 10 μL of 1 mM phosphate buffer at pH 7.4 was added. The final volume of each sample was 200 μL. Samples of each type were prepared in 8 replications.

Quantitative analysis of NTs was conducted by performing a series of excitation-emission matrix (EEM) measurements, where independent 96-well plate was prepared for each bioanalyte. All assays were arranged in a manner analogous to the above-described procedure: appropriate amounts of QDs-GSH solution, phosphate buffer (1 mM, pH 7.4), and NTs were added to each well. The final volume of every sample was 200 μL, with 0.01 mg/mL of QDs-GSH and 0–100 μM of neurotransmitter. All sample types were prepared in 8 replicates.

The 1 mM stock solutions of NTs were prepared daily in 1 mM phosphate buffer at pH 7.4. Prior to EEM acquisition, samples prepared in a 96-well plate were mixed for 1 min. EEMs were generated by recording consecutive emission spectra at decreasing excitation wavelength, starting from 600 nm and going down to 270 nm with a data interval of 10 nm (Table S.1 in Supplementary Information). The range of the recorded emission spectra depended on the excitation wavelength at which the spectrum was acquired: for λ_ex_ ∈ (530 nm, 600 nm), the emission was recorded for λ_em_ ∈ (λ_ex_ + 20 nm, 700 nm), whereas in case of λ_ex_ < 530 nm, the emission was recorded in the range of 550–700 nm. Therefore, the Rayleigh and Raman scattering was not observed in the obtained spectra. All emission spectra were recorded with a resolution of 2 nm. All experiments were performed at room temperature.

### Data analysis

As a result of the performed fluorescence measurements, 34 emission spectra (data vectors) were collected for each sample. Then, the obtained emission spectra were properly ordered in the excitation-emission matrixes, consisting of combinations of all fluorescence intensities obtained for respective wavelengths of excitation and emission (each sample was characterized by 34 × 76 EEM). Prior to chemometric analysis, data vectors corresponding to each emission spectra obtained at next excitation wavelengths were arranged side by side (from emission spectrum at λ_ex_ = 600 nm to emission spectrum at λ_ex_ = 270 nm), so each sample was described by data vector of 1 × 2444 (missing data resulting from the measurement procedure were omitted). The schematic representation of data preparation prior to chemometric analysis is provided in Scheme S.1 in Supplementary Information. The influence of investigated NTs on excitation-emission matrix of QDs-GSH and possibility of their qualitative/quantitative determination was evaluated first by utilizing unsupervised chemometric model–unfolded principal component analysis (UPCA). Unfolded partial least squares–discriminant analysis (UPLS-DA) and unfolded partial Least squarest regression (UPLS) were applied then for identification and quantification of NTs, respectively. Before EEM data modelling, the Savitzky–Golay filter and autoscaling were applied. All preprocessing and chemometric analyses were performed using Solo (Eigenvector Research Inc., Manson, USA), while figures were generated with Origin (OriginLab Corporation, Northhampton, MA, USA) and the MS Excel 2020 (Microsoft, Redmond, USA) software.

## Results and discussion

### Characterization of GSH capped quantum dots

Glutathione as a multifunctional tripeptide, even after being coated on QD shell surface by thiolate moiety, still contains groups of various chemical character, able to bind specific analytes. One amino group and two terminal carboxylate groups might interact through electrostatic forces, whereas numerous regions with different polarity (carbonyl and amide groups) can also contribute to formation of coordination-type bonds or as hydrogen bond donors/acceptors. Therefore, apart from the well-known activity as complexing agent, GSH seems to be an attractive ligand with so far unexplored properties in relation to various types of small-molecule organic compounds.

**Fig. 1:**
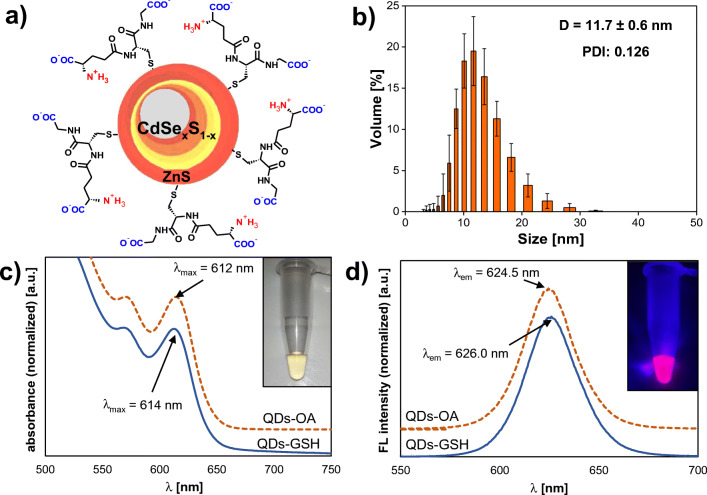
Structure and properties of GSH capped QDs. **a** Schematic illustration of the structure of QDs-GSH. **b** DLS histogram of water-soluble QDs-GSH (*n* = 5; QD concentration 0.1 mg/mL in 1 mM phosphate/NaOH buffer pH 7.4 at 25 °C). **c** Absorption spectra and **d** fluorescence emission spectra of QDs before (dotted line) and after ligand exchange (solid line). Absorption spectra were normalized to the first exciton maxima, and emission spectra were normalized to the emission maxima (wavelengths given within graphs). For clarity, baselines of absorption and emission spectra of QDs-OA were shifted up. Insets show images of QDs-GSH samples (0.1 mg/mL) in **c** visible and **d** UV light

Excitation-emission fluorescence sensing based on cross-affinity of QD surface ligands towards various bioanalytes imposes a number of requirements for the fluorescent nanocrystal. Such non-specific nanosensor must combine very good and stable photoluminescence and the ease of surface functionalization, while maintaining sensitivity to changes of the local chemical environment in the vicinity of nanocrystal’s surface. For this reason, high-quality core-shell quantum dots obtained by organometallic route were utilized in the framework of this work. The proposed approach to QD functionalization using biphasic ligand exchange brings numerous advantages over existing solutions employing one-pot synthesis of GSH-capped nanocrystals. Post-synthetic water-solubilization of highly monodisperse CdSeS cores precoated with ZnS guarantees retention of narrow and sharp emission peaks, which is unattainable for single crystal, cadmium-based QDs [24, 25]. The applied, mild conditions of the ligand exchange process by biphasic phase transfer, without the need for QDs-GSH precipitation or centrifugation, allowed to maintain both good and stable colloidal dispersion in aqueous media and batch-to-batch reproducibility of their optical properties. The scheme of a core-shell quantum dot capped with glutathione (QDs-GSH) prepared in the frame of this work is depicted in Fig. 1a.

As can be seen in Fig. 1c, d, the ligand exchange process had negligibly small influence on the optical characteristics, i.e. the course of the fluorescence absorption and emission spectra. QDs do not show signs of aggregation such as non-specific scattering after ligand exchange compared to the initial nanocrystals dispersed in organic solvent. For both types of examined quantum dots: before (oleic acid-capped) and after modification with GSH, excitonic absorption bands are distinct, while the maximum of the first one undergoes a slight red-shift, from 612 nm to 614 nm (Fig. 1c). Similarly, the emission maximum is slightly shifted (from 624.5 nm for QDs-OA to 626.0 nm for QDs-GSH) due to ligand exchange, while maintaining sharp and narrow emission band (Fig. 1d). The modification and purification of QDs carried out entirely in the liquid phase allowed to avoid typical problems related to fluorescence quenching caused by aggregation of water-soluble quantum dots [27]. In addition, no substantial changes in the position of the absorption and emission maxima were observed between QDs from different batches, and slight differences in the relative fluorescence quantum yields had no effect on the course of the excitation-emission fluorescent fingerprints obtained in the framework of further studies.

The DLS analysis revealed the mean hydrodynamic diameter of QDs-GSH was approximately 11.7 nm (Fig. 1b). The obtained value, only slightly larger than the average diameter of the nanocrystal resulting from TEM analysis (6.0 nm), as well as the lack of visible signs of aggregation reflected in the low polydispersity index (PDI = 0.126), confirms good dispersion and stability of GSH-modified QDs after ligand exchange. Zeta potential measured in 1 mM phosphate/NaOH buffer pH 7.4 was −36.1 ± 0.6 mV, which remains close to the other types of GSH-capped nanocrystals obtained by means of biphasic ligand exchange, which are described in the literature [28]. A strongly negative surface charge of nanocrystals at pH 7.4 can be attributed to deprotonated terminal carboxylate groups of GSH, which make the molecule anionic at pH > pI of the ligand (isoelectric point of ‘free’ glutathione is 5.93 [29]). As can be seen, despite the reported interactions between carboxylate groups and ZnS shell of GSH-capped QDs confirmed by FTIR analysis [30], the contribution of carboxylate moieties on the character of the QD surface remains dominant. As-prepared QDs-GSH, thanks to their stabilization via electrostatic repulsion (zeta potential in the ‘stability window’) and negligible, negative impact of GSH oxidation, turned out to be quite stable in aqueous solution. The samples prepared in this way retained colloidal stability and fluorescent properties for at least 4 weeks. Glutathione turned out to be a much less susceptible to oxidation and desorption QD ligand compared to the simplest zwitterionic capping agent–cysteine [24]. The presented surface properties, stability in aqueous solutions, and attractive photoluminescent properties of as-obtained GSH capped QDs can be considered as a solid foundation for their applications in excitation-emission fluorescence assisted pattern-based sensing.

### Excitation-emission fluorescence response patterns of GSH capped quantum dots

**Fig. 2:**
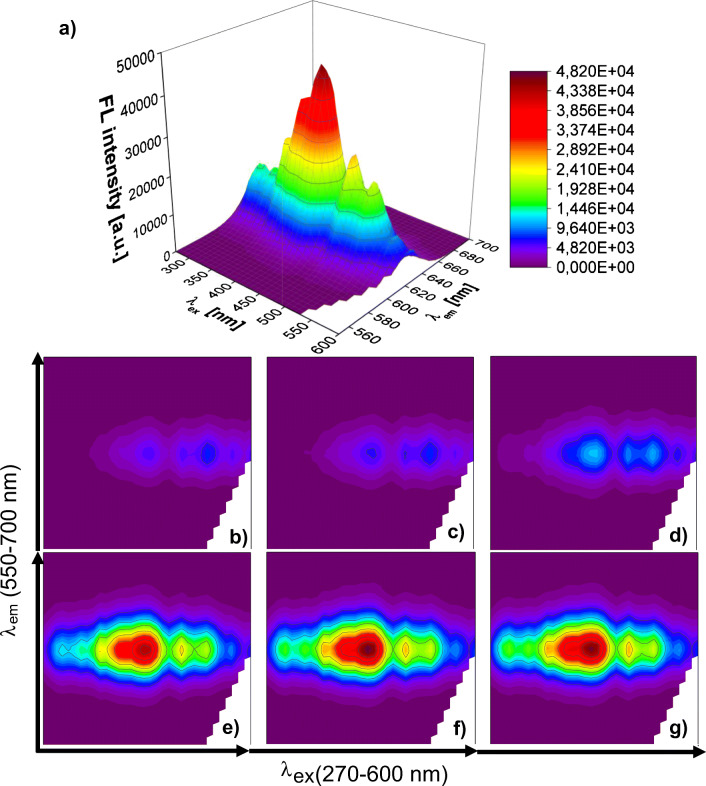
Excitation-emission spectra (λ_ex_ ∈ (270 nm, 600 nm), λ_em_ ∈ (550 nm, 700 nm)) of GSH capped QDs without (**a**) and with (**b–g**) influence of neurotransmitters at 50 μM concentration: **b** dopamine, **c** norepinephrine, **d** epinephrine, **e** serotonin, **f** GABA, and **g** acetylcholine

Excitation-emission spectra of GSH capped QDs before and after the addition of neurotransmitters at 50 μM concentration are shown in Fig. 2. As can be seen, neurotransmitters have affected excitation-emission matrixes (EEMs) of QDs-GSH in two ways: both quenching (Fig. 2b–e) and slight enhancement of fluorescence were observed (Fig. 2f, g). The fluorescence (FL) of QDs-GSH was quenched with different degree by compounds containing a catechol group in their structure (the sequence of quenching intensity is dopamine (Fig. 2b), norepinephrine (Fig. 2c), and epinephrine (Fig. 2d) and to a much lesser extent by serotonin (Fig. 2e). It should be noted that catecholamine NTs caused a change in the fluorescence response over the entire range of the EEM spectrum, while in case of serotonin FL quenching was observed only at emission maximum. In contrast, GABA and acetylcholine caused a slight enhancement of QDs-GSH fluorescence at emission maximum, but the observed effect was similar in both cases (Fig. 2f, g, respectively). The diverse influence of neurotransmitters on the fluorescent properties of QDs-GSH is most likely related to the differences in their chemical structure and thus possibly different kind of interactions between NTs and glutathione surface ligands, as well as the core of QDs. At pH 7.4, dopamine (pKa 9.27), norepinephrine (pKa 8.91), epinephrine (pKa 8.85), and serotonin (pKa 9.31) remain in cationic form [31]; GABA is present as zwitterion [10], and acetylcholine has a positive charge on the nitrogen atom; therefore, electrostatic interaction of all investigated NTs with anionic GSH surface ligands may be possible. However, when considering the structure of these chemical compounds, it seems that only amino and hydroxyl functional groups of dopamine, norepinephrine, epinephrine, and serotonin can participate in the formation of hydrogen bonds with GSH [32]. Moreover, the previous studies revealed that quenching of QD fluorescence by dopamine may be related to its oxidation to dopamine-quinone by ambient O_2_ and therefore an electron-transfer process from QDs to dopamine-quinone species [33, 34]. Since the catecholamines show the greatest stability at acidic pH and could be oxidized to a quinone species both in neutral and alkaline conditions, a similar mechanism is likely for both norepinephrine and epinephrine [31]. It is also well-known fact that glutathione reacts easily with quinone compounds, which might make the possible mechanism of interaction between catecholamine NTs and QDs-GSH more complex [35]. Although the fundamental investigation on the mechanism of interaction of bioanalytes with GSH capped QDs is beyond the scope of this study, a conclusion can be made that the described effects may be responsible for differences in the influence of individual neurotransmitters on excitation-emission fluorescent response of QDs-GSH. It should be emphasized that the observed changes indicate the cross-affinity of GSH capped QDs towards investigated neurotransmitters, which may be the basis for the detection of these compounds in pattern-based sensing approach.

### Identification of neurotransmitters

**Fig. 3:**
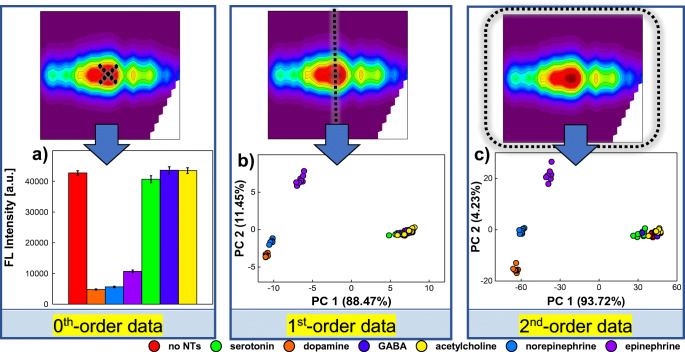
Influence of the order of fluorescent data on the QDs-GSH based discrimination of neurotransmitters (50 μM). **a** Zeroth-order fluorescence data, i.e. fluorescence intensities at λ_ex_ = 430 nm and λ_em_ = 626 nm (mean ± SD, *n* = 8). **b** PCA score plot obtained with first-order fluorescence data, i.e. emission spectra at λ_ex_ = 430 nm and λ_em_ ∈ (550 nm, 700 nm). **c** UPCA score plot obtained with second-order fluorescence data, i.e. excitation-emission spectra at λ_ex_ ∈ (270 nm, 600 nm) and λ_em_ ∈ (550 nm, 700 nm)

Excitation-emission fluorescence fingerprints (EEMs) of GSH capped QDs affected by neurotransmitters were first used as response patterns for the recognition of NTs. However, one of the aims of this work was to verify the impact of type of fluorescent response on the discrimination of model compounds on the basis of non-specific interactions with QDs-GSH. Therefore, we compared neurotransmitters discrimination capability of QDs-GSH using whole EEMs (second-order fluorescent data acquired per sample, 2D) as analytical fingerprints with more commonly used approaches. Thus, when only changes in fluorescence intensity at a specific excitation/emission wavelength (datum, i.e. zeroth-order data acquired per sample, 0D) or at specific excitation wavelength within a certain emission wavelength range were considered (emission spectrum, i.e. first-order data acquired per sample; Fig. 3). To access the sensing capability of glutathione capped QDs towards NTs using different fluorescent data formats, EEMs of QDs-GSH without and with influence of 50 μM NTs were collected first (see “The detection of neurotransmitters” section). Each type of sample was measured in 8 replicates; therefore, a total number of 56 EEMs were obtained (6 neurotransmitters and control samples, each type in 8 replicants). In order to check the influence of the type of fluorescence response on the determination of NTs, zeroth-order (0D) and first-order (1D) fluorescent data were numerically simulated by extraction of adequate signals. In case of zeroth-order data, only changes in the fluorescence intensity at maximum (λ_ex_ = 430 nm, λ_em_ = 626 nm) were extracted from original EEMs. Therefore, mean value and standard deviation of FL intensity for 8 replications of each sample type were calculated and complied to evaluate the possibility of identifying NTs in this approach (Fig. 3a). In case of first- and second-order data, the entire emission spectra at maximum excitation wavelength (λ_ex_ = 430 nm, λ_em_ ∈ (550 nm, 700 nm) see Fig. S.1 in SI) or excitation-emission matrix (34 × 76 data matrix acquired per sample) was analysed, respectively (Fig. 3b, c). Due to the multidimensionality of first- and second-order data, these response patterns were processed by PCA/UPCA, which allows to assess whether the analysed data contains the discriminatory information required for identification purposes [36]. Since chemometric analysis of the second-order data was performed with unfolded EEMs, unfolded PCA model was used [37]. The first-order data were properly arranged in data matrix of 56 × 76 (samples × features) and then subjected to PCA modelling. For the modelling of second-order fluorescent data, EEM representing each sample was unfolded into a vector of 2444 features (see the “Data analysis” section), and then, these unfolded vectors were arranged into a data matrix of 56 × 2444. Results of processing of fluorescence response patterns constructed from emission spectra acquired at λ_ex_ = 430 nm (first-order) and EEM (second-order) data by PCA/UPCA are presented on 2D-PCA score plot in Fig. 3b, c, respectively.

As can be seen in Fig. 3a, the significant quenching of glutathione capped QDs by catecholamine NTs (dopamine, norepinephrine, epinephrine) is clearly visible in zeroth-order fluorescence response and allows for their distinction from control samples (no NTs), as well as non-catecholamine NTs (serotonin, GABA, acetylcholine). However, dopamine and norepinephrine are barely distinguishable from each other, and only epinephrine can be clearly recognized. In addition, neither the slight quenching of fluorescence by serotonin nor the increase in FL intensity caused by GABA and acetylcholine is significant when only changes at maximum λ_ex_/λ_em_ are considered. Therefore, as it could have been suspected, the use of zeroth-order data for the recognition of neurotransmitters based on non-specific interactions with only one type of quantum dots may not be sufficient. However, the positive effect of increasing the order of the fluorescence data on the differentiation of neurotransmitters has been noted (Fig. 3b, c). As shown in Fig. 3b, all of catecholamine NT samples are well separated after processing of first-order fluorescence responses by PCA. However, the use of unfolded EEMs for UPCA modelling appears to be even more beneficial as the clusters of dopamine and norepinephrine are better separated against PC2 (Fig. 3c). Based on the loadings plots on PC2, it can be seen that these differences are most likely due to subtle changes in the baseline for λ_em_ ∈ (550 nm, 600 nm) in case of emission spectrum acquired at λ_ex_ = 430 nm (Fig. S.2.c) and λ_ex_ ∈ (300 nm, 500 nm), λ_em_ ∈ (550 nm, 600 nm) in case of EEM (Fig.S.2.d). We postulate that the observed changes in the baseline might be attributed to the influence of catecholamine NTs on QDs-GSH colloidal stability, i.e. charge-induced agglomeration may have occurred along with the quenching of the quantum dot fluorescence as a result of the interaction of analytes of cationic character (or their functional groups) with the negatively charged surface of QDs-GSH. The deterioration of the electrostatically driven QD repulsion may contribute to QD aggregation reflected in the increased scattering. Interestingly, clusters of serotonin, GABA, and acetylcholine were remarkably similar to control samples (as evidenced by strong overlap of this objects) when only emission spectrum (first-order data) was considered (Fig. 3b), whereas after processing the EEM fingerprints by UPCA, the degree of overlap of these samples decreased (Fig. 3c). In fact, the samples started clustering against PC1 according to the effect that NTs had on the EEMs of QDs-GSH (Fig. 3c; from the right: acetylcholine, GABA—both causing a slight increase of FL intensity at maximum, control samples, and serotonin—quenching the fluorescence of QDs only at maximum). Loadings plots showing the contribution of individual variables to PC1 reveal that the changes of the entire peak in the emission spectra (in case of first-order data, Fig. S.2.a) or whole fluorescent profiles (EEMs; Fig. S.2b) of QDs-GSH have a great influence on the clusterization of NT samples. It should be noted that changes in EEMs (Fig. 2) are not only manifested by the degree of quenching, but also the extent to which this changes occur (that are not visible when we consider only the changes at maximum λ_ex_/λ_em_ or emission spectrum acquired with maximum λ_ex_, see Fig. 3a and Fig. S.1). Therefore, it is not surprising that UPCA modelling of EEM fluorescent fingerprints of QDs-GSH resulted in better separation of the clusters.

**Fig. 4:**
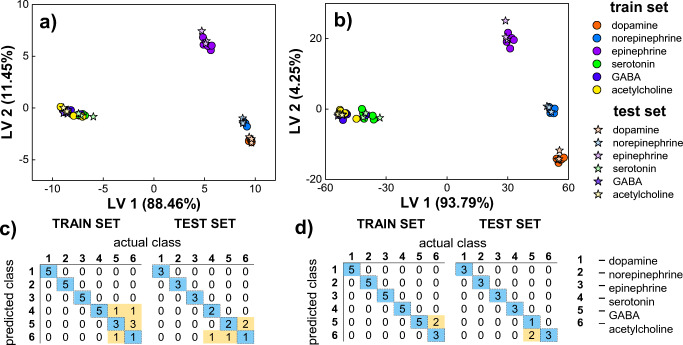
Identification of neurotransmitters by PLS-DA/UPLS-DA models obtained with **a**, **c** first-order and **b**, **d** second-order fluorescence data. **a**, **b** Score plots. **c**, **d** Confusion matrixes

To further evaluate the impact of the type of fluorescence data on the discriminatory power of glutathione capped QDs, first- and second-order fluorescent data were subjected to additional analysis using partial least squares–discriminant analysis (UPLS-DA was used in case of EEM [37]). PLS-DA is a supervised model that allows for the determination of relationship between the response patterns describing individual objects (independent variables) and the class membership of samples (dependent variables). As a result of PLS-DA, variables and directions in the multivariate space are determined (LVs, Latent Variables), which minimize intraclass variance and maximizes interclass variance to differentiate between the response patterns [36]. Prior to PLS-DA/UPLS-DA analysis, data matrixes previously applied for PCA/UPCA were randomly divided into train and test set (62.5% and 37.5% of all data set, respectively; control samples were excluded). Thus, train matrixes of 30 × 76 and 30 × 2444 were applied for the establishment of PLS-DA/UPLS-DA models, while test matrixes of 18 × 76 and 18 × 2444 were used for validation (for first- and second-order data, respectively). The number of the LVs for each model was determined based on the minimalization of RMSECV (Root Mean Square Error of Cross-Validation) by performing cross-validation of venetian blind. The two most significant LVs have been used to generate the PLS-DA score plots, showing clustering of objects from both train and test sets (Fig. 4a, b). In order to compare the quality of developed models, 4 quality performance metrics were calculated (accuracy, sensitivity, precision, specificity; see Table S.2 in SI) on the basis of confusion matrices (Fig. 4c, d) that provided numbers of true negatives TN, false negatives FN, false positives FP, and true positives TP [36]. The decision on class affinity was made on the basis of the ‘most probable’ rule, which means that the sample was assigned to that class which the highest value in the predicted output vector was obtained. Since application of big amount of data from EEM measurements might lead to model overfitting [38], additional parameters of RMSE (Root Mean Square Error of Calibration) and RMSEP (Root Mean Square Error of Prediction) were also taken into account to better access and compare generalization capabilities of the obtained models (Table 1).

**Table 1:** Quality performance parameters of PLS-DA/UPLS-DA models applied for neurotransmitter identification using first- and second-order fluorescence data

1st order fluorescence data										
	**Train set**				**Test set**				**RMSE**	**RMSEP**
	**Accuracy**	**Precision**	**Sensitivity**	**Specificity**	**Accuracy**	**Precision**	**Sensitivity**	**Specificity**		
Dopamine	80.00%	1.000	1.000	1.000	77.78%	1.000	1.000	1.000	0.259	0.259
Norepinephrine		1.000	1.000	1.000		1.000	1.000	1.000	0.312	0.314
Epinephrine		1.000	1.000	1.000		1.000	1.000	1.000	0.064	0.072
Serotonin		0.714	1.000	1.000		1.000	0.667	0.923	0.340	0.343
GABA		0.500	0.600	0.913		0.500	0.667	0.923	0.330	0.328
Acetylcholine		0.500	0.200	0.852		0.333	0.333	0.867	0.330	0.328
Mean		0.786	0.800	0.960		0.806	0.779	0.952	0.273	0.274
2nd order fluorescence data										
	**Train set**				**Test set**				**RMSE**	**RMSEP**
	**Accuracy**	**Precision**	**Sensitivity**	**Specificity**	**Accuracy**	**Precision**	**Sensitivity**	**Specificity**		
Dopamine	93.33%	1.000	1.000	1.000	88.89%	1.000	1.000	1.000	0.083	0.099
Norepinephrine		1.000	1.000	1.000		1.000	1.000	1.000	0.117	0.113
Epinephrine		1.000	1.000	1.000		1.000	1.000	1.000	0.038	0.033
Serotonin		1.000	1.000	1.000		1.000	1.000	1.000	0.110	0.102
GABA		0.714	1.000	1.000		1.000	0.333	1.000	0.295	0.299
Acetylcholine		1.000	0.600	0.926		0.600	1.000	1.000	0.291	0.288
Mean		0.952	0.933	0.988		0.933	0.888	1.000	0.156	0.156

As can be seen in Fig. 4, modelling both first-order (1D) and second-order (2D) fluorescent data by PLS-DA/UPLS-DA allowed for perfect identification of compounds that contains a catechol group in their structure, i.e. dopamine, norepinephrine, and epinephrine. Thus, catecholamine NTs can be determined using QDs-GSH with precision, sensitivity, and specificity equal 1.000 (Table 1), regardless the type of fluorescence data considered. It is worth noting that application of EEM fingerprints for UPLS-DA modelling resulted in significant minimization of RMSE and RMSEP values (Table 1), which is not surprising given better separability of their clusters visible in the UPLS-DA score plot (Fig. 4c). The performance of classification models differs in percent of correct classification (accuracy) achieved, which in case of first-order fluorescence data was 80.00% for train and 77.78% for test set, while 93.33% and 88.89% of samples were correctly classified when whole EEMs were applied for model development (for train and test set, respectively; Table 1). The confusion matrixes reveal that these differences in quality of NT identification are related to different ability to discriminate among non-catecholamine neurotransmitters (serotonin, GABA, acetylcholine) depending on the type of fluorescence response under consideration (Fig. 4c, d). When only first-order data were used, objects representing serotonin, GABA, and acetylcholine were misclassified (among themselves) in case of both train and test sets (Fig. 4c), which in consequence results in unsatisfactory precision, sensitivity, and specificity of the identification of these compounds (Table 1). It is not surprising given the significant overlap of these samples that can be seen in the PLS-DA score plot (Fig. 4a). In contrast, application of EEM fingerprints for the development of UPLS-DA model allowed for ideal identification of serotonin (precision, sensitivity, and specificity were 1.000 for both train and test set; Table 1). Additionally, even though GABA and acetylcholine clusters were not ideally separated (Fig. 4b) and some misclassification occurred (acetylcholine was incorrectly classified as GABA in train set, while GABA as acetylcholine in test set; Fig. 4d), their identification is more satisfactory in terms of precision, sensitivity, and specificity for both train and test set (comparing to the model built on the basis of emission spectra at maximum λ_ex_; Table 1). The only exception is GABA, whose samples from the test set were identified with poor sensitivity of 0.333. It should be emphasized that UPLS-DA model developed using unfolded EEMs did not show tendency to overfitting, i.e. the results obtained for the independent test set are comparable to those achieved during calibration stage (Table 1). These results demonstrate that excitation-emission fluorescence assisted QDs-based sensing has the potential to expand the pool of bioanalytes so far detectable with the use of QDs and is a perfect candidate for various pattern-based sensing approaches.

### Quantitative detection of neurotransmitters

When the possibility of qualitative analysis of neurotransmitters was confirmed, the proposed methodology was also extended to quantitative analysis of NTs by means of unfolded partial least squares regression. To probe the ability of the developed sensing system in quantitative analysis strategy, a series of excitation-emission fluorescence measurements were carried out applying various concentrations of NTs (0, 1, 2.5, 5, 10, 20, 30, 40, 50, 60, 80, and 100 μM; see “The detection of neurotransmitters” section). As a result, a total number of 576 EEMs (6 NTs × 12 concentrations × 8 replicates of each sample type) were obtained and subjected to further data analysis. Before performing a quantitative analysis using unfolded PLS, EEMs were processed with the use of unfolded PCA to visualize the effect of NT concentration on clusterization of the samples. Prior to UPCA modelling, each EEM of size 34 × 76 was unfolded into a vector of 1 × 2444 size (see “Data analysis” section). For each of the investigated bioanalytes, i.e. 6 NTs, the final matrix of size 96 × 2444 was applied for UPCA analysis (12 concentration levels, 8 replicants).

**Fig. 5:**
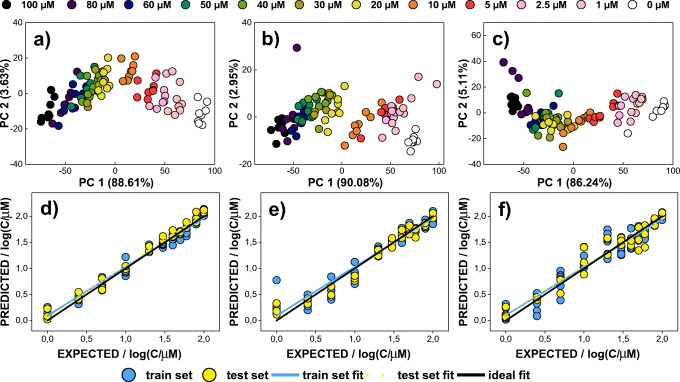
Quantitative analysis of catecholamine neurotransmitters. Differentiation of samples at various concentration levels visible on UPCA score plots for **a** dopamine, **b** norepinephrine, and **c** epinephrine. UPLS model performances shown as linear fit of the predicted vs. real logarithm of concentrations for **d** dopamine, **e** norepinephrine, and **f** epinephrine

**Fig. 6:**
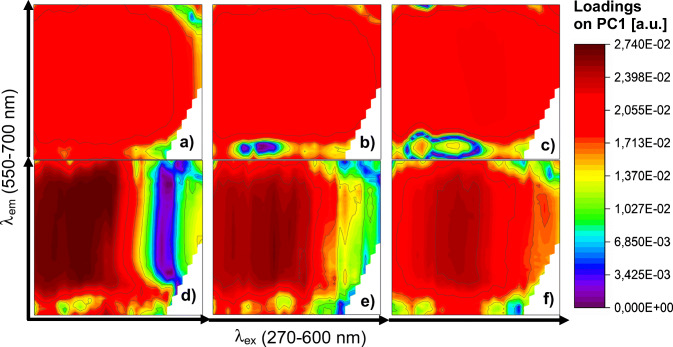
Refolded loadings on the first principal component (PC1) obtained for excitation-emission spectra of various concentrations of **a** dopamine, **b** norepinephrine, **c** epinephrine, **d** serotonin, **e** GABA, and **f** acetylcholine. For clarity, the absolute values of the loadings were presented

As can be seen in Fig. 5a–c, samples of catecholamine neurotransmitters (dopamine, norepinephrine, epinephrine) at various concentrations are clustering in similar manner. All controls (0 μM) are easily discernible from samples with NT addition (scores on PC1 ≈ 100). As a concentration of bioanalyte increases, the value of PC1 scores decreases, which results in clustering of samples of the same concentration. In addition, samples with lower concentrations (0–10 μM) are characterized by PC1 > 0 and better separation of the clusters, while samples containing 20 μM or higher concentration of catecholamine NTs are exhibited by PC1 < 0 and stronger overlap of representatives of different classes. Therefore, a linear relationship between the fluorescence response pattern of QDs-GSH and logarithm value of catecholamine NT concentration was established (Fig. S.3). However, in case of neurotransmitters that do not contain a catechol group in their structure (serotonin, GABA, acetylcholine), the influence of their concentration on EEM of GSH capped QDs was negligible (Fig. S.4a-c). Although samples containing only QDs-GSH are clearly separated from the samples with addition of non-catecholamine NTs, no significant clustering related to bioanalyte content was observed. Interestingly, the loadings plots reveal that different ranges of excitation-emission spectrum are the most relevant in PC1 for individual NTs (Fig. 6). Due to the fact that the interaction of non-catecholamine neurotransmitters with QDs-GSH is most probably of a different nature than in case of catecholamine NTs and therefore their impact on EEM fluorescence fingerprints of QDs-GSH is much smaller, this is a result that could be expected.

Quantitative analysis of neurotransmitters was realized by means of unfolded PLS. Prior to UPLS modelling and estimation of model performance, the data matrix (consisting of unfolded EEM data) previously applied for UPCA was split randomly into train and test set (62.5% and 37.5% of all data set, respectively; controls were excluded). The optimal number of LVs that characterized each model was chosen based on the minimalization of RMSECV. The model performance was characterized after linear fitting of the predicted data (UPLS predicted values of logarithm of NT concentration) to the expected data (logarithm of the real concentration of NTs). Hence, regression parameters of the linear fit (‘a’, ‘b’, ‘*R*^2^’) were calculated for the train and test objects, assuming that in ideal model slope (‘a’) and determination coefficient (‘*R*^2^’) are both equal 1, whereas intercept (‘b’) is 0. In addition, the parameters of RMSE and RMSEP were also considered to assess the quality of the obtained models (Table 2, Table S.3). The established UPLS models confirm the ability of the developed system to quantify catecholamine neurotransmitters (dopamine, norepinephrine, epinephrine). As can be seen in Fig. 5d–f, linear regression parameters were satisfactory in case of all three catecholamine NTs, for both train and test set. Regression parameters for train data were close to the ideal ones, i.e. slope on the level of 0.922–0.970, intercept of 0.040–0.102, and determination coefficients was 0.900–1.000 (Table 2). Moreover, the results obtained for external test sets prove great generalization capabilities of the developed models, as evidenced by similar values of ‘a’ (0.913–1.002), ‘b’ (0.014–0.121), and ‘*R*^2^’ (0.935–0.987) in relation to linear fit parameters characterizing train sets (Table 2). Low values of the RMSE and RMSEP additionally indicate that the obtained models are not prone to overfitting, and excitation-emission fluorescence assisted pattern-based sensing with QDs-GSH can be successfully used for quantitative analysis of catecholamine NTs (Table 2). As it would be expected from the results of the UPCA analysis (Fig. S.4a-c), the UPLS models developed for non-catecholamine neurotransmitters confirm that their quantitative analysis in the considered concentration range using the developed system is hardly possible (Fig. S.4d-f, Table S.3). These results show that although excitation-emission fluorescence spectroscopy may be a useful technique to identify compounds based on their non-specific interactions with quantum dot, obtaining the information required for quantification using only one type of QDs might be challenging or even limited to specific compounds.

**Table 2:** Parameters of linear fit of real and UPLS-predicted logarithms of concentration (μM) for catecholamine neurotransmitters

	**Dopamine**	**Norepinephrine**	**Epinephrine**
**RMSE**	0.051	0.116	0.158
**RMSEP**	0.088	0.124	0.160
	**Train set**		
**a**	0.970 ± 0.020	0.934 ± 0.039	0.922 ± 0.037
**b**	0.040 ± 0.028	0.095 ± 0.055	0.102 ± 0.052
***R***^**2**^	0.979 ± 0.091	0.916 ± 0.180	0.922 ± 0.171
	**Test set**		
**a**	1.002 ± 0.021	0.971 ± 0.035	0.913 ± 0.043
**b**	0.048 ± 0.029	0.014 ± 0.049	0.121 ± 0.061
***R***^**2**^	0.987 ± 0.074	0.962 ± 0.124	0.935 ± 0.155

## Conclusions

In this study, glutathione capped QDs were utilized in excitation-emission fluorescence assisted pattern-based sensing, for both qualitative and quantitative detection of neurotransmitters. The proposed detection strategy was based on the cross-affinity of QDs-GSH towards neurotransmitters of different chemical structure, evidenced by their diverse impact on QD EEM fluorescence fingerprints. The presented work shows that application of excitation-emission fluorescence spectroscopy allows to capture subtle differences in the impact of neurotransmitters on fluorescent properties of QDs-GSH that are not visible when only changes in FL at one λ_ex_/λ_em_ (zeroth-order data) or emission spectrum acquired at maximum λ_ex_ (first-order data) are considered. It must be underlined that this is our another work [15] which shows that the use of excitation-emission fluorescence spectroscopy could possibly extend the sensing capability of QD pattern-based systems developed in the future. Moreover, utilization of whole fluorescence profiles (EEM) of nanoreceptor may simplify the sensing element applied for the recognition of bioanalytes by providing analyte-specific multidimensional optical information. The proposed excitation-emission QDs-GSH assay was also successfully used for the quantification of catecholamine neurotransmitters (dopamine, norepinephrine, epinephrine) at micromolar concentration range. However, the results obtained for non-catecholamine NTs (serotonin, GABA, acetylcholine) revealed that quantitative analysis using only one type of QD may be restricted to specific compounds, which is definite limitation of presented method.

It must be underlined that this work shows only the proof of principle that EEM fluorescence response of QDs provides significant improvement in comparison to zeroth-order or first-order fluorescence response. For detailed picture of the performance of the proposed sensing strategy, following research must be undertaken. As the most important ones, we consider studying of limits of the proposed method—i.e. its selectivity and possible interferences, with further tests showing applicability for mixtures of analytes and real samples. Analytical performance characterization in the terms of LOD and LOQ should be also performed, with special emphasis given to the extension of the method to nanomolar concentration range. Finally, even better potential of this sensing strategy could be achieved with the application of data analysis dedicated to second-order data structure, namely n-way PLS or PARAFAC.

## Supplementary information

ESM 1 (PDF 611 kb)
