# Supplementary material for: Excitation-emission fluorescence matrix acquired from glutathione capped CdSeS/ZnS quantum dots in combination with chemometric tools for pattern-based sensing of neurotransmitters
Source: Mikrochim Acta. 2021 Sep 15;188(10):343. doi: 10.1007/s00604-021-04984-x (PMC8443496; doi:10.1007/s00604-021-04984-x)
Supplement: Supplementary file 1 — (PDF 611 kb) [file 604_2021_4984_MOESM1_ESM.pdf]

# Electronic Supplementary Material

# Excitation-emission fluorescence matrix acquired from glutathione capped CdSeS/ZnS quantum dots in combination with chemometric tools for pattern-based sensing of neurotransmitters

**Klaudia Głowacz[1]*, Marcin Drozd[1,2], Patrycja Ciosek-Skibińska[1]***

[1] Chair of Medical Biotechnology, Faculty of Chemistry, Warsaw University of Technology, Noakowskiego 3, 00-664

Warsaw, Poland

[2] Centre for Advanced Materials and Technologies CEZAMAT, Poleczki 19, 02-822, Warsaw, Poland

*Correspondence: pciosek@ch.pw.edu.pl (PC-S); kglowacz@ch.pw.edu.pl (KG)

## Table of contents

**Scheme S. 1.** .......... **2**
**Table S. 1.** .......... **3**
**Fig S. 1.** .......... **4**
**Fig S. 2.** .......... **5**
**Table S. 2.** .......... **6**
**Fig S. 3.** .......... **7**
**Fig S. 4.** .......... **8**
**Table S. 3.** .......... **9**

**Scheme S.1.** Schematical representation of procedure for Excitation-Emission Fluorescence data preparation. **(a)** Preparation of the Excitation-Emission Matrixes by arranging the appropriate emission spectra acquired during fluorescence measurements. **(b)** Arrangement of the Excitation-Emission Matrixes in a cube and their unfolding by combining the spectral modes.

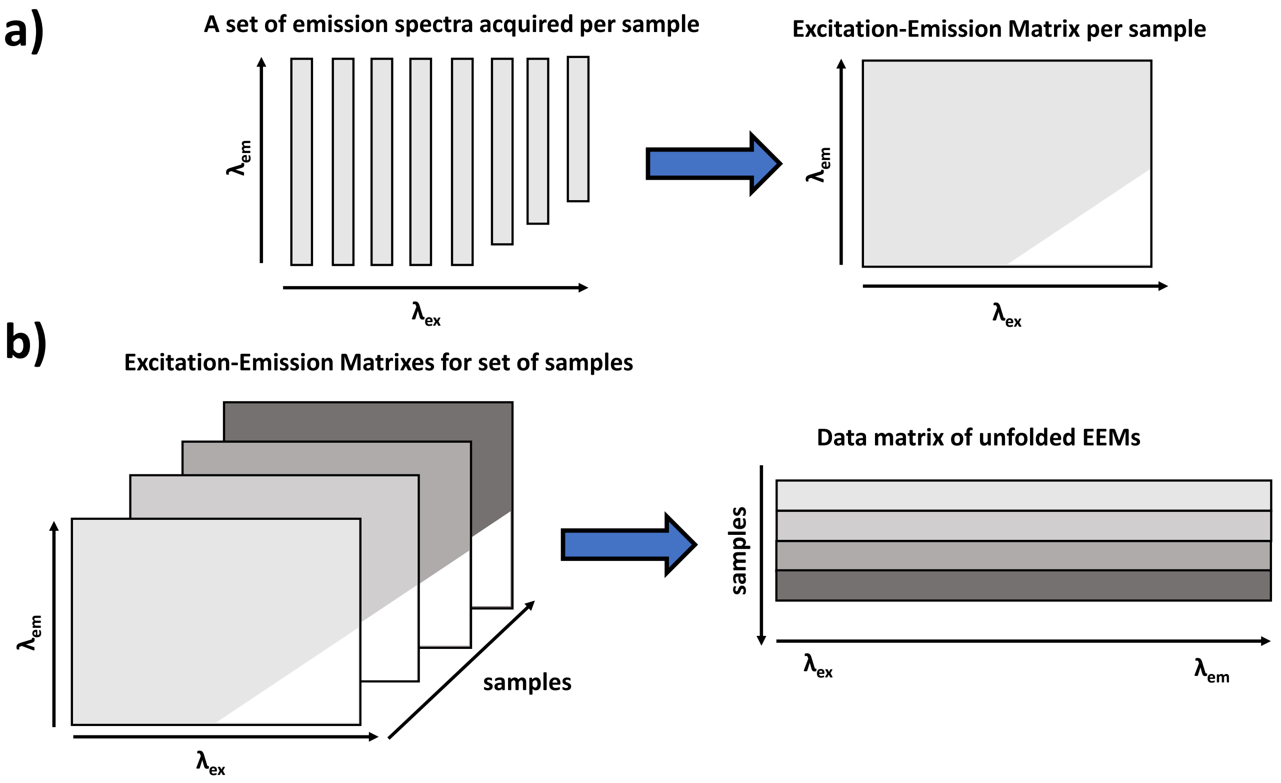

**Table S.1.** The measurement protocol used for acquisition of Excitation-Emission Matrixes.

| Procedure step | $\lambda_{ex}$ [nm] | $\lambda_{em}$ [nm] |
|---|---|---|
| **1** | 600 | 620 – 700 |
| **2** | 590 | 610 – 700 |
| **3** | 580 | 600 – 700 |
| **4** | 570 | 590 – 700 |
| **5** | 560 | 580 – 700 |
| **6** | 550 | 570 – 700 |
| **7** | 540 | 560 – 700 |
| **8** | 530 | 550 – 700 |
| **9** | 520 | 550 – 700 |
| **10** | 510 | 550 – 700 |
| **11** | 500 | 550 – 700 |
| **12** | 490 | 550 – 700 |
| **13** | 480 | 550 – 700 |
| **14** | 470 | 550 – 700 |
| **15** | 460 | 550 – 700 |
| **16** | 450 | 550 – 700 |
| **17** | 440 | 550 – 700 |
| **18** | 430 | 550 – 700 |
| **19** | 420 | 550 – 700 |
| **20** | 410 | 550 – 700 |
| **21** | 400 | 550 – 700 |
| **22** | 390 | 550 – 700 |
| **23** | 380 | 550 – 700 |
| **24** | 370 | 550 – 700 |
| **25** | 360 | 550 – 700 |
| **26** | 350 | 550 – 700 |
| **27** | 340 | 550 – 700 |
| **28** | 330 | 550 – 700 |
| **29** | 320 | 550 – 700 |
| **30** | 310 | 550 – 700 |
| **31** | 300 | 550 – 700 |
| **32** | 290 | 550 – 700 |
| **33** | 280 | 550 – 700 |
| **34** | 270 | 550 – 700 |

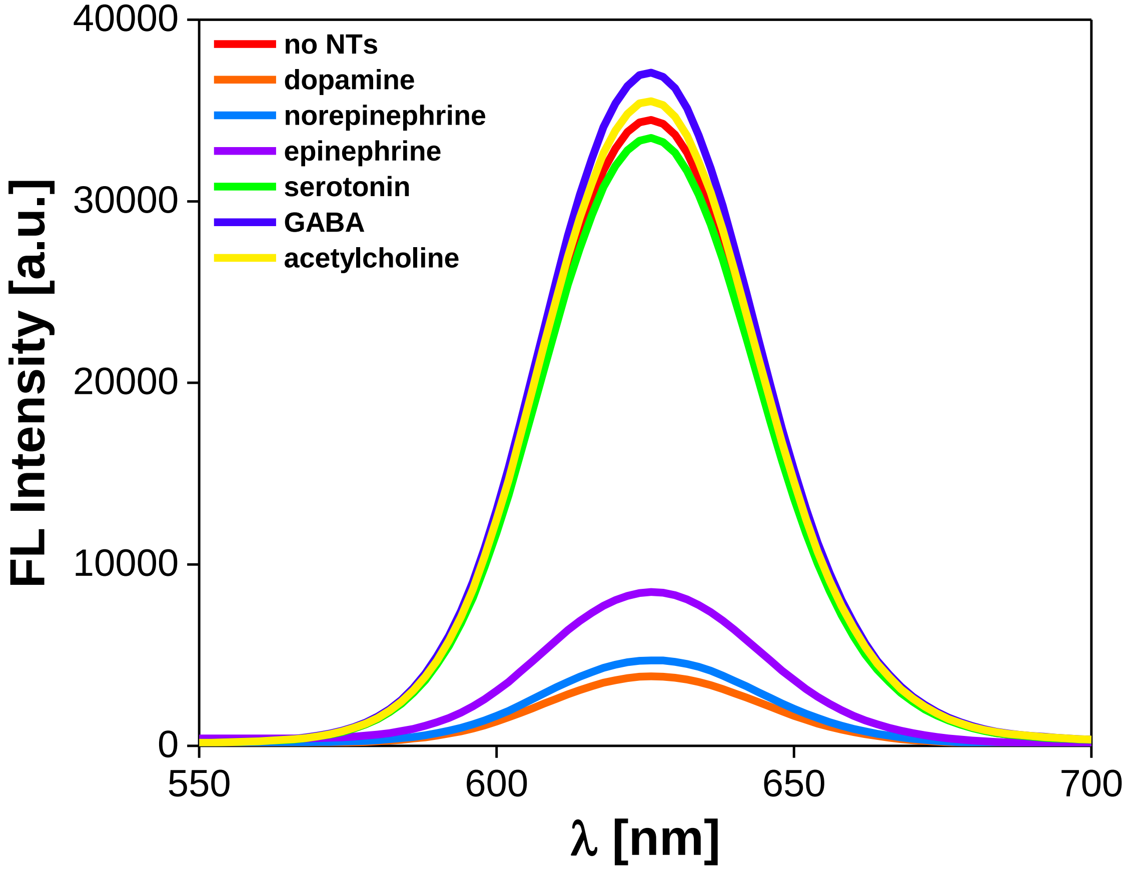


**Fig S. 1.** Fluorescence spectra (i.e. 1st-order fluorescence data) of GSH capped QDs without and with influence of neurotransmitters at 50 μM concentration acquired at $\lambda_{ex}$ = 430 nm, $\lambda_{em}$ ∈ [550 nm, 700 nm].

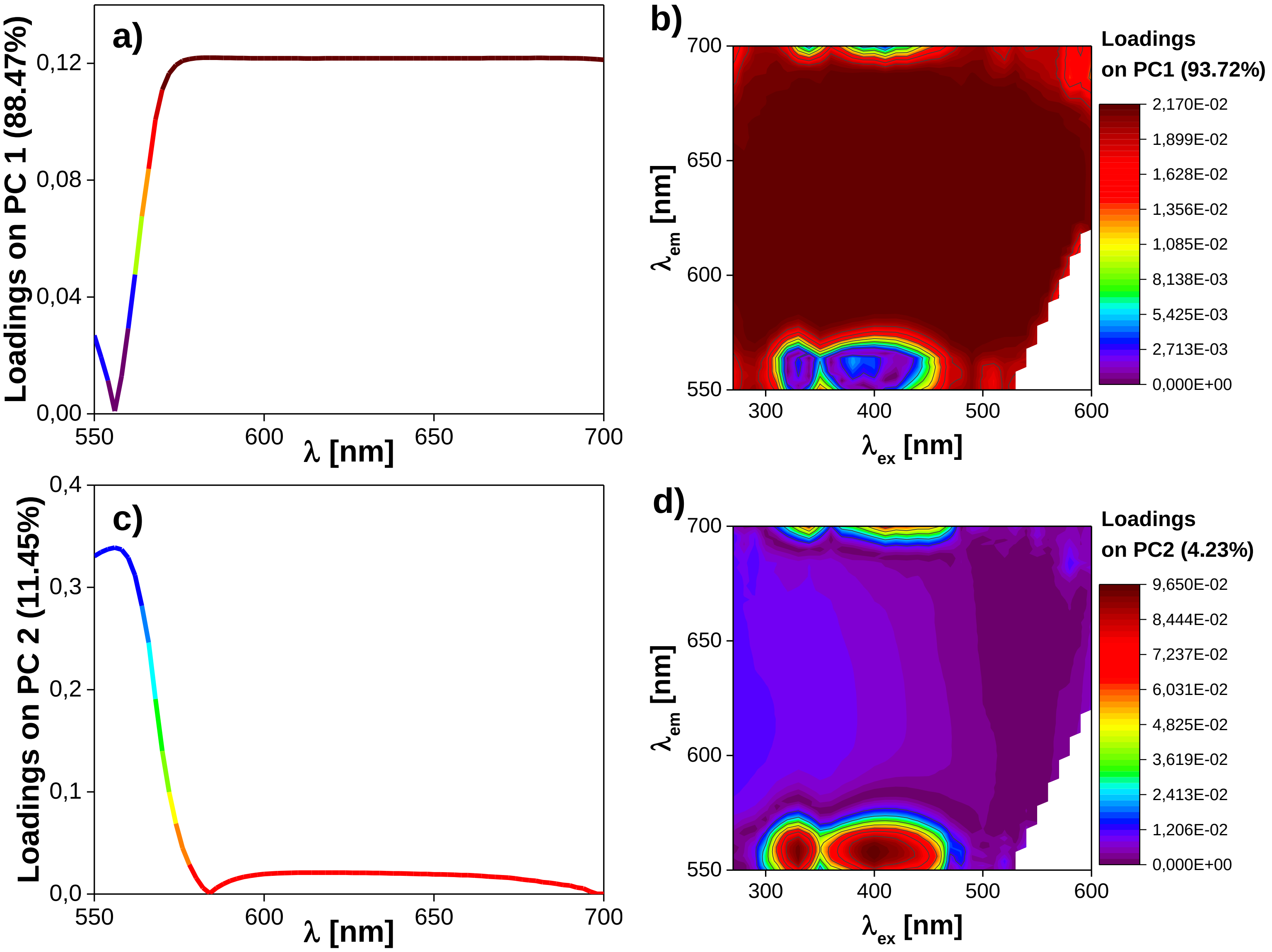


**Fig S. 2.** Loadings plots on **(a, b)** PC1 and **(c, d)** PC2 showing which variables correspond to clusterization of samples in PCA/UPCA models obtained with **(a, c)** 1st-order fluorescence data (i.e. emission spectra acquired at $\lambda_{ex} = 430$, $\lambda_{em} \in$ [550 nm, 700 nm] and **(b, d)** 2nd-order fluorescence data (i.e. Excitation-Emission Matrix acquired at $\lambda_{ex} \in$ [270 nm, 600 nm], $\lambda_{em} \in$ [550 nm, 700 nm]. For clarity, the absolute values of the loadings were presented.

**Table S. 2.** Definitions of quality performance metrics used to assess the quality of PLS-DA/UPLS-DA models **(a)** accuracy, **(b)** precision, **(c)** sensitivity, **(d)** specificity. TN – true negatives, FN – false negatives, FP – false positives, TP – true positives.

**a)**

$$accuracy = \frac{(TP + TN)}{(TP + TN + FP + FN)}$$

**b)**

$$precision = \frac{TP}{(TP + FP)}$$

**c)**

$$sensitivity = \frac{TP}{(TP + FN)}$$

**d)**

$$specificity = \frac{TN}{(TN + FP)}$$

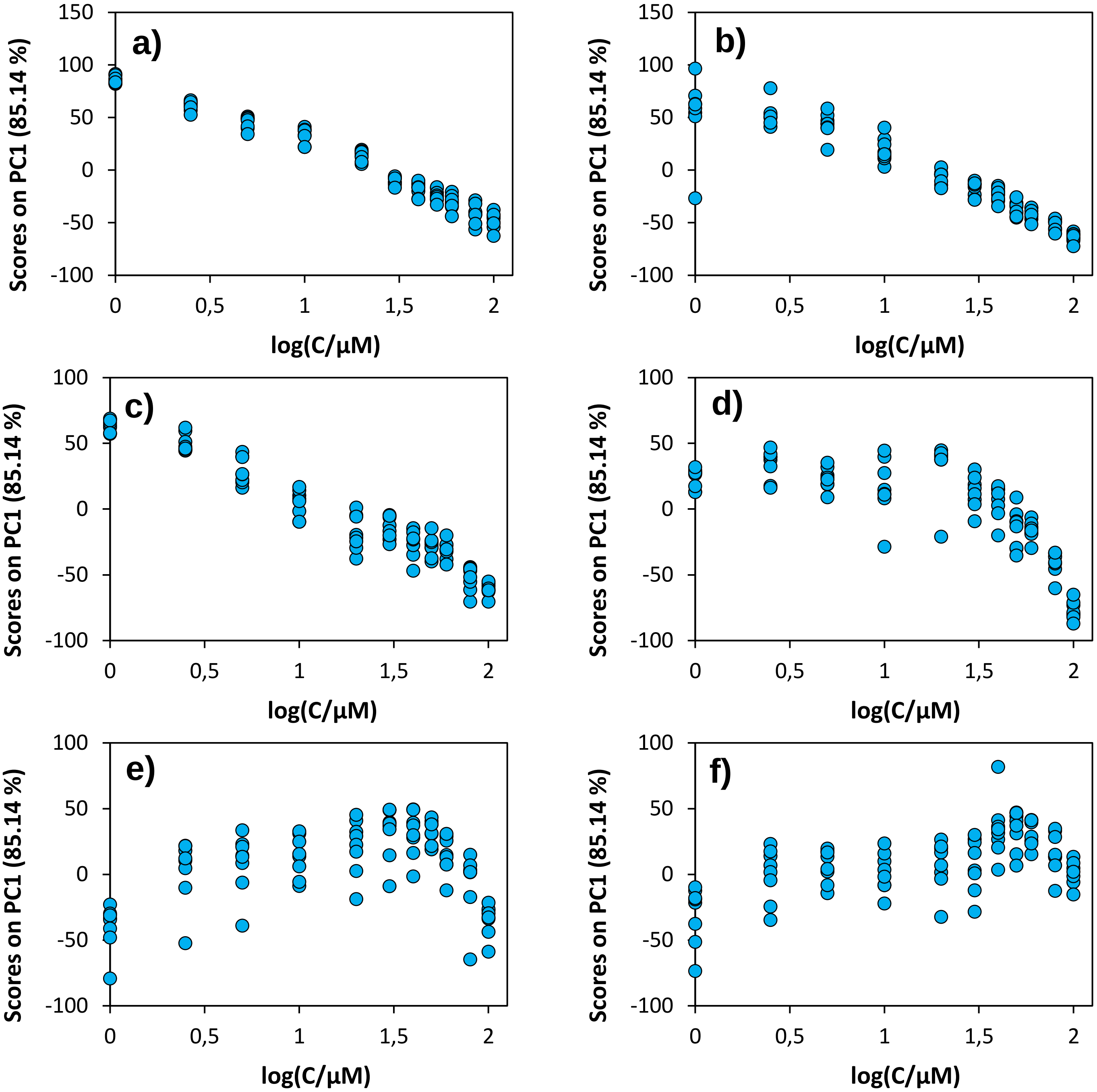


**Fig S. 3.** Relationship between the logarithms of concentration (µM) of neurotransmitters and scores on PC1 for **(a)** dopamine**, (b)** norepinephrine, **(c)** epinephrine, **(d)** serotonin, **(e)** GABA, **(f)** acetylcholine.

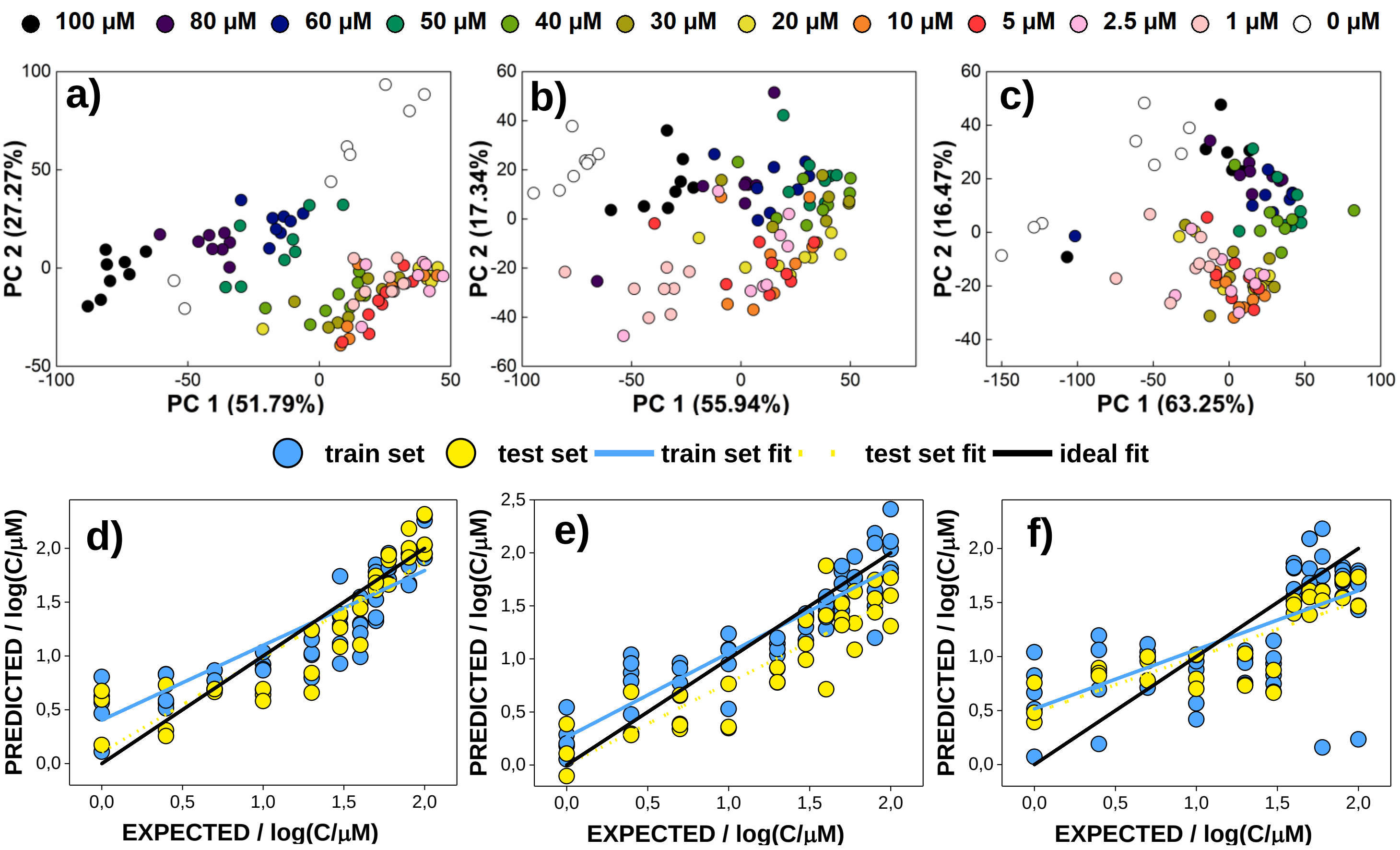


**Fig S. 4.** Quantitative analysis of catecholamine neurotransmitters. Differentiation of samples at various concentration levels visible on UPCA score plots for **(a)** serotonin, **(b)** GABA, **(c)** acetylcholine. UPLS model performances shown as linear fit of the predicted vs. real logarithm of concentrations for **(d)** serotonin, **(e)** GABA, **(f)** acetylcholine.

**Table S. 3.** Parameters of linear fit of real and UPLS-predicted logarithms of concentration (μM) for non-catecholamine neurotransmitters.

| | serotonin | GABA | acetylcholine |
|---|---|---|---|
| RMSE | 0.093 | 0.071 | 0.277 |
| RMSEP | 0.289 | 0.404 | 0.409 |
| | | train set | |
| a | 0.695 ± 0.078 | 0.7946 ± 0.052 | 0.547 ± 0.093 |
| b | 0.404 ± 0.110 | 0.2614 ± 0.073 | 0.516 ± 0.130 |
| R2 | 0.602 ± 0.360 | 0.817 ± 0.240 | 0.397 ± 0.429 |
| | | test set | |
| a | 0.881 ± 0.079 | 0.773 ± 0.072 | 0.515 ± 0.069 |
| b | 0.106 ± 0.111 | 0.001 ± 0.101 | 0.482 ± 0.097 |
| R2 | 0.800 ± 0.284 | 0.791 ± 0.257 | 0.641 ± 0.249 |
